# Three drugs vs two drugs first-line chemotherapy regimen in advanced gastric cancer patients: a retrospective analysis

**DOI:** 10.1186/s40064-015-1545-y

**Published:** 2015-12-01

**Authors:** Alessandro Bittoni, Michela Del Prete, Mario Scartozzi, Mirco Pistelli, Riccardo Giampieri, Luca Faloppi, Maristella Bianconi, Stefano Cascinu

**Affiliations:** Medical Oncology, AOU Ospedali Riuniti-Università, Politecnica Delle Marche, 60126 Ancona, Italy; Oncologia Medica, Azienda Ospedaliero-Universitaria di Cagliari, Presidio Policlinico D. Casula, Strada St. 554, KM 4.500, Cagliari, 09042 Monserrato, CA Italy

**Keywords:** Advanced gastric cancer, First-line chemotherapy, Three drugs, Two drugs

## Abstract

The definition of the standard chemotherapy regimen for advanced gastric cancer is still a matter of debate. Aim of our analysis was to retrospectively assess whether an intensive, three-drugs, front line approach could be comparable to a sequential (two-drugs front line then second line) in terms of RR (response rate), PFS (progression free survival) and OS (overall survival) in advanced gastric cancer patients in the clinical practice. Patients with metastatic gastric cancer who have received a first-line combination chemotherapy with a two or three-drugs regimen were included in our analysis. We divided our patients into two groups, A and B, based on the first line chemotherapy administered (group A = three drugs; group B = two drugs). A total of 425 patients were eligible for our analysis. 216 patients (50.8 %) received three chemotherapeutic agents (group A) and 209 patients (49.2 %) received a two drugs regimen as first-line combination chemotherapy (group B). RR for group A and B was 44 and 29.6 %, respectively (p = 0.0005), median PFS was 7.3 months in group A and 4.5 months in group B (p = 0.0007). No significant difference was found in terms of OS. The addition of a third drug to a doublet chemotherapy regimen appeared more active in terms of response rate and PFS. However median OS resulted comparable. On this basis, the use of a sequential approach may represent a reasonable strategy for patients unwilling or unable to undergo a more intensive treatment without compromising OS.

## Background

Gastric cancers remains one of the leading causes of cancer mortality worldwide even thought its incidence has been decreasing in recent years. Radical surgical resection still represents the only potentially curative treatment. Unfortunately, more than half of gastric carcinomas are diagnosed in an advanced stage, when resection is no longer possible. In this setting chemotherapy is still the main treatment option for patients with advanced disease. Median overall survival (OS) of 8–12 months has been reported in patients undergoing chemotherapy compared with 3–5 months for those treated with best supportive care alone (Glimelius et al. 1997). Several drugs such as fluorouracil (5-FU), capecitabine, cisplatin, oxaliplatin docetaxel, epirubicin, paclitaxel and irinotecan are major components of conventional regimens. S-1 and mytomicin C are also being used for the treatment of gastric cancer. Combination chemotherapy has been shown to be associated with a statistically significant (p = 0.001) survival benefit compared to monotherapy in a meta-analysis of several clinical trials (Wagner et al. 2006). This corresponded to a small but clinically relevant 1-month mean average survival benefit. This meta-analysis also showed that including anthracyclines in a 5-FU-cisplatin combination had a modest survival advantage over cisplatin-5-FU alone (HR 0.77). Finally, the meta-analysis also showed that three-drug combinations had a significant survival benefit compared to two-drug combinations. Several clinical trials (Van Cutsem et al. 2006; Ajani et al. 2007a, b; Cunningham et al. 2008) investigating first-line therapy in advanced gastric cancer suggested that a triplet chemotherapy regimen might have a survival benefit over doublets but the evidence is not fully convincing since these results are mostly dependent on older studies. Adding more chemotherapeutic agents seems to confer more benefit but at the same time inevitably adds toxicity, thus to date, a triplet chemotherapy combinations is not an established global standard as yet. Besides, in the daily clinical practice, administering a three-drugs treatment may prove difficult in advanced gastric cancer patients, who often present with multiple co-morbidities and poor performance status. Consequently in this scenario a doublet with a fluoropyrimidine and platinum is still an acceptable alternative and remains the cornerstone of gastric cancer treatment. On this basis, the definition of the standard chemotherapy regimen for advanced gastric cancer remains a matter debate.

Aim of our analysis was to retrospectively assess whether an intensive, three-drugs, front line approach could be comparable to a sequential (two-drugs front line then second line) in terms of RR (response rate), PFS (progression free survival) and OS (overall survival) in advanced gastric cancer patients.

## Patients and methods

### Patients selection

The study population was selected from a central database including patients with gastric cancer treated and followed at our institution. Patients with histologically confirmed, recurrent or metastatic gastric or gastroesophageal junction adenocarcinoma who have received a first-line combination chemotherapy with a two or three-drugs chemotherapy regimen were included in our analysis. Patients were eligible if they had measurable or evaluable metastatic disease; Eastern Cooperative Oncology Group performance status 0–2; age ≥18; no central nervous system metastasis. Patients treated with trastuzumab or were excluded from our analysis.

We divided patients into two groups, A and B, based on the characteristics of the first line chemotherapy combination used (group A = three drugs; group B = two drugs). The chemotherapy regimen administered to each patient was chosen by the treating physician, mainly according to patient’s characterstics, such as age, performance status, comorbidities and organ function.

This analysis was approved by Ethical committee AOU Ospedali Riuniti, Umberto I of our institution.

### Response to treatment

Physical examination, complete blood counts and biochemical tests were carried out before each cycle of therapy. A chest and abdomen CT scan was performed every four cycles and when disease progression was clinically suspected to document the extent of disease and to evaluate the response to treatment. The response was assessed using the Response Evaluation Criteria in Solid Tumours (RECIST) 1.0.

### Statistical analysis

For statistical analysis, OS and PFS were defined, respectively, as the interval between the first day of first-line chemotherapy until the time of the first occurrence of progression, death from any cause or to the date of last follow-up visit and as the interval between the first day of first-line chemotherapy to the date of death or to the date of the last follow-up visit. Analyses of PFS and OS curves were performed using the Kaplan–Meier method. We compared the response rates between the two groups using the Chi-square test.

## Results

### Characteristics of the patients

A total of 425 advanced gastric cancer patients treated with chemotherapy were included in our analysis. Males and females were 63 and 37 % respectively with a median age of 64 years (range 29–84 years). The two groups of patients resulted comparable for most of baseline characteristics of clinical relevance (age, sex, ECOG PS, site of metastases, tumour histology, previous surgical resection, peritoneal diffusion, as shown in Table 1. The majority of the patients (76 %) had previously undergone surgery for their disease. Peritoneal tumour diffusion was present in 215 cases (50.6 %). Tumour involved gastroesophageal junction in 18 % of cases. After disease progression, about 45 % of patients received a second-line chemotherapy.

**Table 1:** Patients’ characteristics

	Group A (3 drugs)	Group B (2 drugs)	p
Age			
Median (range), years	61 (29–79)	68 (32–84)	
Sex			
M	138	128	0.64
F	78	81	
ECOG PS			
0–1	165	155	0.67
2	51	54	
Histotype			
Intestinal	127	133	0.36
Diffuse	89	76	
Site of primary tumor			
Gastroesophageal junction	34	43	0.24
Stomach	182	166	
Surgery			
Yes	180	143	0.0005
No	36	66	
Neoadjuvant/Adjuvant chemotherapy			
Yes	74	62	
No	142	147	0.36
Metastatic site			
1	129	134	0.18
≥2	87	75	
Peritoneal carcinosis			
Yes	118	97	0.9
No	98	112	
Second line chemotherapy			
Yes	92	99	0.37
No	124	110	

### Treatment

Two-hundred and sixteen patients (50.8 %) received three chemotherapeutic agents as first-line treatment (group A); chemotherapy included a platinum derivate and a fluoropyrimidine with the addition of an anthracycline (about 70 %), or mytomicin C (23 %) or docetaxel (5.5 %); a minority of patients (2 %) received a platinum-free combination (FAM regimen) as shown in Table 2. Two-hundred and nine patients (49.2 %) received a two drugs regimen as first-line combination chemotherapy (group B); 199 patients received a fluorouracil based chemotherapy with the addition of a second drug such as a platinum derivate (62 %), mytomicin C (27 %), irinotecan (6.7 %). Ten patients received a chemotherapy regimen which not contained fluorouracil. A total of 191 (44.9 %) patients received second line chemotherapy. The percentage of patients receiving second line chemotherapy was found to be slightly higher in group B compared to group A (47.4 vs 42.6 %, p = 0.37). FOLFIRI (5-fluorouracil, leucovorin and irinotecan) was the most commonly used regimen in this setting and 43.5 % of all patients who received second line chemotherapy received this combination. Other second line treatments included taxanes (paclitaxel or docetaxel), FOLFOX (5-fluorouracil, leucovorin and oxaliplatin) and combinations of 5-fluorouracil and mytomicin C.

**Table 2:** First-line chemotherapy regimens administered in the two groups of patients

Group A	N. patients	Group B	N. patients
PELF	138 (64 %)	CDDP + 5-FU	86 (41 %)
ECF	12 (5.5 %)	FOLFOX	43 (21 %)
CDDP + 5-FU + MMC	50 (23 %)	5-FU + MMC	56 (27 %)
TCF	12 (5.5 %)	FOLFIRI	14 (6.7 %)
FAM	4 (2 %)	CDDP + docetaxel	9 (4 %)
		IROX	1 (0.3 %)

PELF (cisplatin, epirubicin, 5-FU and leucovorin), ECF (epirubicin, cisplatin and 5-FU), TCF (docetaxel, cisplatin, 5-FU), MMC (mytomicin C), FAM (5-FU, adriamycin, cyclofosfamid) CDDP (cisplatin), FOLFIRI (5-FU, leucovorin, irinotecan, irinotecan), IROX (irinotecan, oxaliplatin)

### Efficacy

**Fig. 1:**
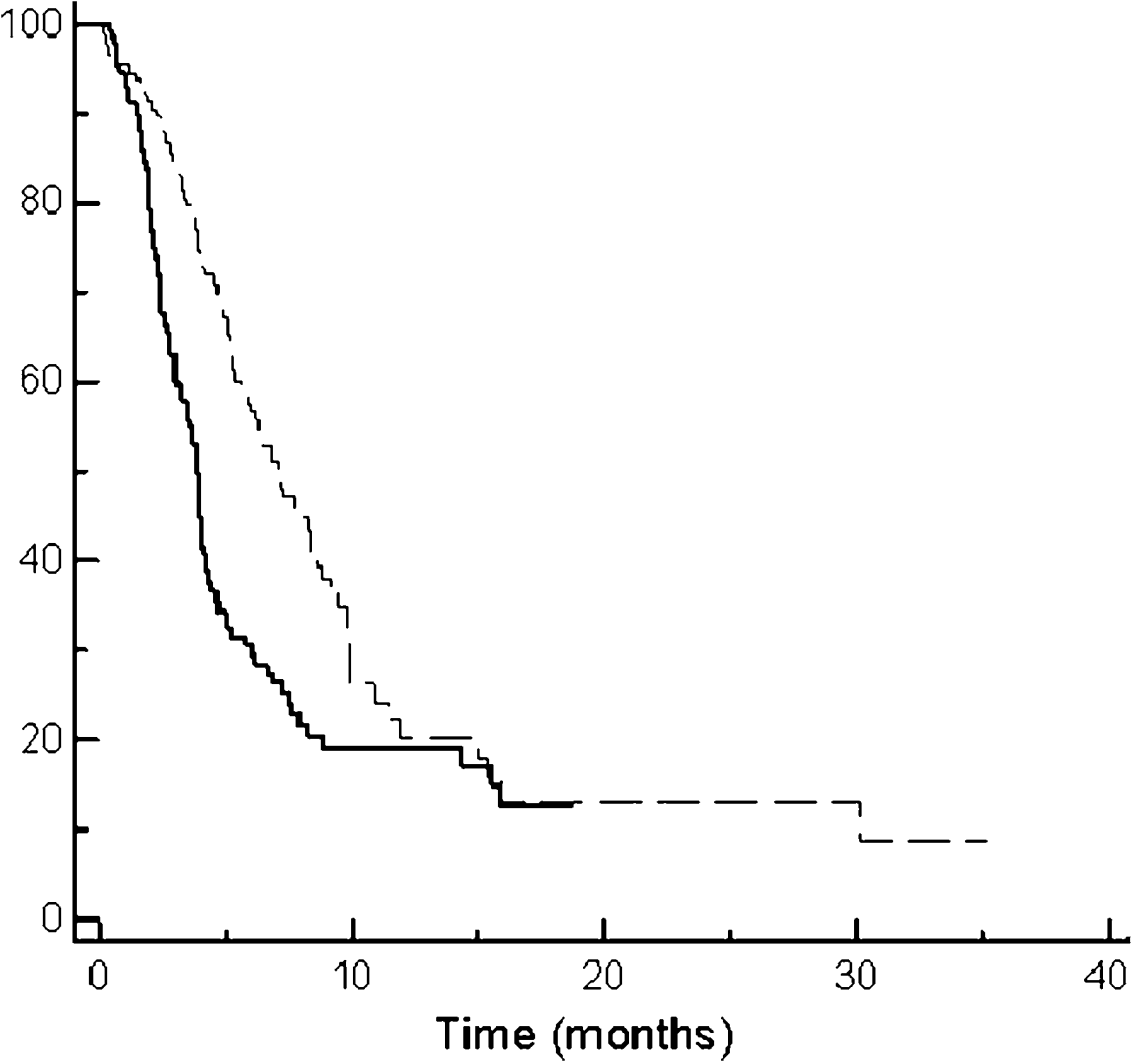
Kaplan-Meier curves for median progression-free survival (PFS) of advanced gastric cancer patients in group A (three-drugs first-line chemotherapy) (*dotted line*) and group B (two-drugs first-line chemotherapy) (*thin line*) (7.3 vs. 4.5 months, p = 0.0007)

**Fig. 2:**
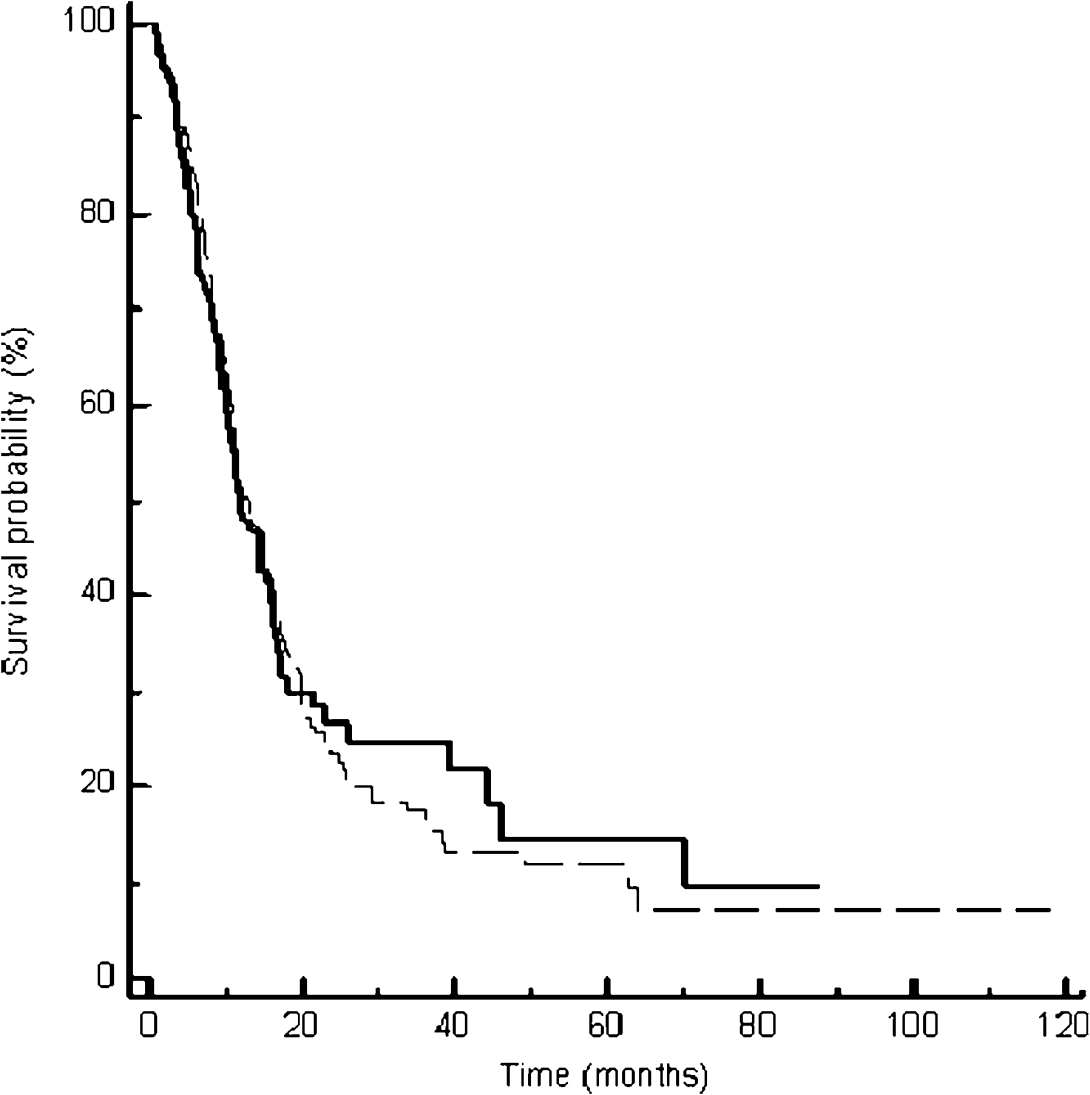
Kaplan-Meier curves for median overall survival (OS) of advanced gastric cancer patients in group A (three-drugs first-line chemotherapy) (*dotted line*) and group B (two-drugs first-line chemotherapy) (*thick line*) (13 vs. 11.8 months, p = 0.84)

In 387 patients with measurable disease, we observed 4 (2 %) complete responses and 82 (42 %) partial responses in group A and 3 complete responses (1.5 %) and 54 (28.1 %) partial responses in group B. 52 (26.7 %) and 56 (29.3 %) patients achieved a stable disease as best response to first-line treatment in group A and B, respectively (Table 3). The overall response rate observed in patients treated with a three-drugs first-line chemotherapy (group A) was 44 %, significantly higher compared to response rate in group B (29.6 %), p = 0.0005. The outcome of patients treated with a first-line three drugs regimen was better also in terms of PFS, with a median PFS of 7.3 months in group A compared to 4.5 months in group B, p = 0.0007 (Fig. 1). Overall Survival did not differ significantly between the two groups, with a median OS of 13 months for group A and of 11.8 months for group B, p = 0.84 (Fig. 2).

**Table 3:** Response rate in patients with measurable disease treated with three vs two drugs first-line chemotherapy

Response rate	Group A (3 drugs; n.pts 195)	Group B (2 drugs; n.pts 192)	
CR	4 (2 %)	3 (1.5 %)	
PR	82 (42 %)	54 (28.1 %)	
ORR (CR + PR)	86 (44 %)	57 (29.6 %)	p = 0.005
SD	52 (26.7 %)	56 (29.3 %)	
PD	57 (29.3 %)	79 (41.1 %)	

_*CR* complete response, *PR* partial response, *SD* stable disease, *PD* progressive disease, *ORR* overall response rate_

### Toxicity

Most common adverse events to first-line chemotherapy are listed in Table 4. Grade 3–4 neutropenia was more common in patients treated with three drugs regimens (group A) compared to group B (43 vs 28 %) and also incidence of febrile neutropenia was higher in group A. Severe fatigue was found to be more common in group A (28 %) compared to group B (15 %) of patients. Other common treatment’s related toxicities were nausea and vomiting, diarrhea and peripheral neuropathy.

**Table 4:** Most common adverse events to first-line chemotherapy in the two groups of patients

Adverse event	Group A		Group B	
	All grade (%)	Grade 3–4 (%)	All grade (%)	Grade 3–4 (%)
Anemia	75	15	72	9
Neutropenia	78	43*	65	28
Trombocytopenia	18	6	21	7
Febrile neutropenia		19*		8
Nausea/vomiting	67	16	61	14
Diarrhea	42	10	36	8
Stomatitis	35	7	29	6
Fatigue	71	28*	66	15
Peripheral neuropathy	36	14	28	8

_NCI-CTC toxicity criteria 1.0_

_* Statistically significant differences (p < 0.05) for comparison between group A and group B_

## Discussion

Standard chemotherapy for advanced gastric cancer patients represents a matter of debate among the oncology community. Although a recent meta-analysis demonstrated a survival advantage for the use of three-drugs first-line regimens, a two drugs, cisplatin-based chemotherapy is still considered a preferred choice in many countries for both the daily practice and as reference arm in randomized trials.

In fact, the apparent small survival advantage and the toxicity profile linked to triplets chemotherapy regimens made the option of doublets regimens more attractive in this setting.

Our retrospective analysis suggested that a three-drugs first-line chemotherapy may be superior in terms of response rate and PFS to a two-drugs regimen in the treatment of advanced gastric cancer patients. Nevertheless, no significant difference in OS was demonstrated between the two treatments.

These results are in contrast with the findings from a few randomized trials demonstrating a benefit for three-drug regimens versus traditional doublets. A modest benefit from the addition of an anthracycline to a two-drugs platinum-based treatment has been demonstrated by meta-analyses (Wagner et al. 2006). Nevertheless, no randomized trial has confirmed this benefit and most of the trials have actually compared different three drugs regimen, as FAMTX, containing fluorouracil, doxorubicin and methotrexate, vs ECF (epirubicin, cisplatin and infusional fluorouracil) (Webb et al. 1997) or ECF vs the combination of mytomicin, cisplatin and fluorouracil (MCF) (Ross et al. 2002).

The addition of taxanes to a platinum and 5-FU combination has also been shown to improve the efficacy of treatment in gastric cancer. The V325 trial randomized 445 advanced gastric cancer patients to docetaxel, cisplatin and 5-FU (TCF) or cisplatin and 5-FU (CF) (Van Cutsem et al. 2006). The trial showed that adding docetaxel to CF significantly improved response rate (37 vs 25 %, p = 0.01) and prolonged Time To Progression (5.6 months; 95 % CI 4.9–5.9; vs 3.7 months; 95 % CI 3.4–4.5) and Overall Survival (9.2 months; 95 % CI 8.4–10.6; vs 8.6 months; 95 % CI 7.2–9.5). These results demonstrated a small but significant advantage in overall survival for a first-line three-drugs regimen compared to a doublet but they also showed an expected increase in treatment’s toxicity. For example, in the V325 trial, the treatment with DCF resulted in an higher frequency of grade 3 or 4 toxic effects, such as neutropenia (82 vs 57 %), febrile neutropenia (29 vs 12 %), diarrhoea (19 vs 9 %), and lethargy (19 vs 14 %) compared to the CF group. Our study confirmed these results, showing an higher incidence of toxicities, such as neutropenia and fatigue, in the group of patients treated with three drugs regimens. Nevertheless, other studies have evaluated different schedules of taxanes-based three-drugs regimens, showing that tolerability of these treatments may be improved with the use of weekly schedule () Tebbutt NC1, Cummins MM, Sourjina T et al. Randomised, non-comparative phase II study of weekly docetaxel with cisplatin and 5-fluorouracil or with capecitabine in oesophagogastric cancer: the AGITG ATTAX trial. Br J Cancer 2010).

We hypothesized that the lack of a significant difference in overall survival between patients treated with three or two drugs in first line, observed in our analysis, may be related to the influence of second line treatment in the outcome of patients. Increasing evidences support the use of a salvage treatment after progression to first-line chemotherapy, but in the oldest phase III trials the use of a subsequent second-line treatment was restricted only to a small percentage of patients. A pooled analysis including 1080 patients, from three phase III trials of first-line chemotherapy for advanced gastric cancer, showed that 20 % of patients went on to receive a second line chemotherapy with a 13 % response rate and a progression-free survival of 5.6 months (Chau et al. 2004). Also in the more recent REAL-2 study only 14 % of the patients received a second line treatment after progression.

Recently, a small phase III randomized trial evaluated single-agent irinotecan compared to best supportive care in pre-treated advanced gastric cancer patients (Thuss-Patience et al. 2011). The trial was closed early due to poor accrual, after the enrolment of 40 patients. The study demonstrated a statistically significant overall survival benefit for irinotecan over best supportive care alone, with a median OS of 4.0 months for the irinotecan arm versus 2.4 months for the best supportive care arm (p = 0.012, HR = 0.48, 95 % CI 0.25–0.92). Similar results were achieved by another randomized phase III trial comparing second line treatment with either docetaxel or irinotecan to best supportive care in pretreated advanced gastric cancer patients (Parket al. 2011). Among the 202 randomized patients, a significant benefit in OS (5.1 vs 3.8 months; p = 0.004) was observed for second-line chemotherapy over best supportive care. Another recent trial evaluated docetaxel in second line treatment of advanced gastric cancer and demonstrated a benefit in overall survival for chemotherapy compared to active symptoms control (Ford et al. 2014).

In our study the use of salvage treatment was well balanced between the two groups of patients. FOLFIRI was the most used regimen in the second line setting. Therefore, our results seem to suggest that the sequential use of active agents, in advanced gastric cancer, may be equivalent to a more aggressive, simultaneous use of the drugs in terms of overall survival. The comparison of these different approaches has been already evaluated in different gastrointestinal cancers. In particular, in advanced colorectal cancer, two randomized trials, CAIRO and FOCUS showed that staged strategies of a single agent fluoropyrimidine followed by a doublet combination are not inferior to up-front doublets in terms of overall survival (Koopman et al. 2007; Seymour et al. 2007). More recently, the use of a sequential strategy has been assessed also in the treatment of advanced gastric cancer in a phase II trial with encouraging results (Loupakis et al. 2010). In this study, the authors evaluated a sequential strategy involving three different chemotherapy doublets containing cisplatin, irinotecan and docetaxel in combination with infused fluorouracil and folinic acid. The results obtained in terms of PFS and OS (6.8 and 11.1 months respectively) were comparable to those observed in trials using three-drugs first-line regimens with a favourable safety profile.

However, given our observations suggesting an improved response rate and progression-free survival with triplets, we could speculate that this therapeutic option may represent a first choice in patients with tumour related symptoms or bulky disease, where the chances to use a second-line treatment are less probable and a prompt disease control is needed.

Considering the less favourable toxicity profile of a three-drugs first-line treatment, this option should be chosen after a careful assessment of patient’s performance status and comorbidities, keeping in mind that maintaining the patient’s quality of life is a primary goal of treatment in metastatic setting. Indeed, in our analysis an high rate of febrile neutropenia was seen in patients treated with three-drug combination therapy. The retrospective nature of the study is one of the limitations of this analysis with possible selection bias, together with the heterogeneity of chemotherapy regimens used, including some outdated or unusual regimens. Moreover, after the recent demonstration of efficacy of the anti-HER2 agent trastuzumab in the treatment of HER-2 positive advanced gastric cancer (Bang et al. 2010), the combination of trastuzumab with a chemotherapy doublet (cisplatin with a fluoropyrimidine) represents the treatment of choice in this subset of patients.

Further prospective clinical trials, evaluating different chemotherapy sequences in the treatment of advanced gastric cancer, are warranted to improve patients’ outcome and minimize the toxicity of treatment.
